# Exemestane potency is unchanged by common nonsynonymous polymorphisms in CYP19A1: results of a novel anti‐aromatase activity assay examining exemestane and its derivatives

**DOI:** 10.1002/prp2.313

**Published:** 2017-04-27

**Authors:** Amity Peterson, Zuping Xia, Gang Chen, Philip Lazarus

**Affiliations:** ^1^ Laboratory of Philip LazarusDepartment of Pharmaceutical SciencesWashington State UniversitySpokaneWashington

**Keywords:** Anti‐aromatase activity, aromatase inhibitor, breast cancer, dihydroexemestane, exemestane, pharmacogenetics, polymorphism

## Abstract

Exemestane (EXE) treats estrogen receptor positive (ER+) breast cancer in postmenopausal women by inhibiting the estrogen‐synthesizing cytochrome P450 CYP19A1. Variability in the severity and incidence of side effects as well as overall drug efficacy may be partially explained by genetic factors, including nonsynonymous variation in CYP19A1, also known as aromatase. The present study identified phase I EXE metabolites in human liver microsomes (HLM) and investigated mechanisms that may alter the extent of systemic estrogen deprivation in EXE‐treated women with breast cancer, including whether functional polymorphisms in aromatase cause differential inhibition by EXE and whether EXE metabolites possess anti‐aromatase activity. The potency of EXE and ten of its derivatives was measured with HEK293‐overexpressed wild type aromatase (CYP19A1*1) using a rapid novel UPLC tandem mass spectrometry method. Of the ten compounds assayed, five were poor inhibitors (IC_50_ ˃ 50 *μ*mol/L) of wild type aromatase while five others, including the major metabolite, 17*β*‐dihydroexemestane (17*β*‐DHE), exhibited moderate potency, with IC_50_ values ranging between 1.2 and 7.1 *μ*mol/L. The anti‐aromatase activity of EXE was also tested with two common allozymes, aromatase^Thr201Met^ (CYP19A1*3) and aromatase^Arg264Cys^ (CYP19A1*4). Differential inhibition of variant aromatase is unlikely to account for variable clinical outcomes as EXE‐mediated inhibition of aromatase^Thr201Met^ (IC_50_ = 0.86 ± 0.12 *μ*mol/L) and aromatase^Arg264Cys^ (IC_50_ = 1.7 ± 0.65 *μ*mol/L) did not significantly differ from wild type (IC_50_ = 0.92 ± 0.17 *μ*mol/L). Although less potent than the parent drug, these results suggest that active metabolites may contribute to the therapeutic mechanism of EXE.

**Abbreviations**
17*α*‐DHE: 17*α*‐dihydroexemestane
17*β*‐DHE: 17*β*‐dihydroexemestane
6‐HME: 6‐Hydroxymethylandrosta‐1,4,6‐triene‐3,17‐dione
AAA: anti‐aromatase activity
AI: aromatase inhibitor
AKRs: aldo‐keto reductases
CBR1: carbonyl reductase 1
CYP450: cytochrome P450
ER+: estrogen receptor positive
EXE: exemestane
GC‐MS: gas chromatography coupled to mass spectrometry
HLM: human liver microsomes
NMR: nuclear magnetic resonance spectroscopy
UGT: UDP‐glucuronosyltransferase
UPLC/MS/MS: ultra‐performance liquid chromatography coupled to tandem mass spectrometry

## Introduction

Exemestane is a synthetic androgen prescribed to postmenopausal women with ER+ breast cancer (Coombes et al. 2007; Paridaens et al. 2008). As an adjuvant endocrine therapy, EXE irreversibly inhibits the aromatase‐mediated production of estrogens from androgen precursors, a process known as aromatization (Hong et al. 2007). A previous pharmacokinetics study found that the maximum plasma concentration of EXE in postmenopausal women with a prior history of breast cancer ranged from 3.0 to 15.6 ng/mL following 2 weeks of oral dosing (25 mg/day) while the maximum amount of its 17*β*‐DHE metabolite varied 7‐fold with reported values of 0.22–1.58 ng/mL (Traina et al. 2008).

Prescriptive information states that EXE is extensively metabolized, in part by aldo‐keto reductases (AKRs) (Pfizer, 2016). A key phase I metabolic pathway of EXE is C17 reduction to form a hydroxyl moiety vulnerable to phase II conjugation and excretion (Sun et al. 2010). Recent studies independently confirmed that five purified hepatic cytosolic reductases, AKRs 1C1‐4 and carbonyl reductase 1 (CBR1), reduce EXE to the active metabolite 17*β*‐DHE (Platt et al. 2016). Formation of 17*α*‐dihydroexemestane (17*α*‐DHE), a novel metabolite with unknown anti‐aromatase activity (AAA), was catalyzed by AKR1C4 and CBR1 (Platt et al. 2016). A second metabolic pathway in human liver preparations is C6 exomethylene oxidation by CYP3A4 to form multiple secondary metabolites (Pfizer, 2016). The chemical structures of the C6‐oxidized metabolites, as well as detailed information regarding their capacity to inhibit aromatase are omitted from the product leaflet dispensed with EXE tablets (Pfizer, 2016).

Several studies imply that EXE hepatic metabolism may be more complex than previously believed with possibly undiscovered metabolites and coaction by additional cytochrome P450s (CYP450s) (Kamdem et al. 2011; Platt et al. 2016). Comprehensively identifying phase I EXE metabolites is warranted, because EXE derivatives may contribute to systemic estrogen blockade through aromatase inhibition. The presence of 17*β*‐DHE as a major metabolite in human plasma has been unequivocally confirmed in studies of postmenopausal women taking EXE (Evans et al. 1992; Traina et al. 2008). However, past attempts to identify less‐studied metabolites have been speculative due to the lack of standard reference compounds. Using GC‐MS, three peaks likely corresponding to C6‐oxidized metabolites were detected in the urine of healthy male volunteers (Cavalcanti Gde et al. 2011). Another study found six metabolites, including 17*β*‐DHE, in human urine following administration of radiolabeled EXE (Cocchiari et al. 1994). However, both studies of urinary EXE metabolites were hampered by a lack of comparison of physiochemical properties between the suspected metabolites and known standards. Six possible metabolite peaks were observed in human liver microsomes presented with EXE substrate (Kamdem et al. 2011). One peak was confirmed to be 17*β*‐DHE and another was tentatively designated as 6‐hydroxymethylandrosta‐1,4,6‐triene‐3,17‐dione (6‐HME) (Kamdem et al. 2011). The identities of the remaining four peaks could not be established (Kamdem et al. 2011).

The current study addresses methodological issues that have historically undermined phase I EXE metabolite identification. First, a reference library of C6 and C17‐modified EXE analogs was synthesized to confirm the identity of suspected metabolites observed in incubations of EXE with human liver microsomes. Secondly, a newly developed UPLC/MS/MS method eliminates the need for organic extraction to remove residual substrate prior to analysis unlike previous scintillation‐based studies of AAA (Thompson and Siiteri 1974). Instead, low levels of estrone formation are quantitated directly rather than extrapolated from tritiated water release during the aromatization of radiolabeled androstenedione. Interestingly, aromatase from human placental microsomes is used in traditional AAA screenings (Thompson and Siiteri 1974). CYP1A1 is well‐expressed in human placenta and extensively metabolized EXE in an in vitro assay using recombinant baculosome‐expressed CYP450s (Kamdem et al. 2011; Uhlén et al. 2015). Therefore, background phase I metabolism in human placental microsomes may complicate the analysis of AAA assays. However, expression analysis has shown that HEK293 are CYP450 and UDP‐glucuronosyltransferase (UGT)‐null (data not shown). To circumvent potential confounding from endogenous enzymes in placental preparations, aromatase‐overexpressing HEK293 were created in the present study to evaluate the potency of EXE analogs in impeding estrogen biosynthesis.

While it is well‐accepted that genetic differences may influence an individual's drug disposition for many pharmaceuticals, the extent to which polymorphisms in aromatase explain interindividual variation in EXE potency is unclear. Interestingly, aromatase has several common nonsynonymous variants which might contribute to variability in drug disposition by altering its affinity for EXE (Ma et al. 2005), potentially affecting EXE efficacy or toxicity risk. Consequently, we also compared the efficacy of EXE in inhibiting two allozymes, aromatase^Thr201Met^ and aromatase^Arg264Cys^ relative to the wild type enzyme.

## Materials and Methods

### Materials

Hangzhou DayangChem Co. (Hanzhou City, China) supplied the androgens boldenone, testosterone, and 4‐andostene‐3,17‐dione for the synthesis of EXE and its analogs. Tokyo Chemical Industry Co. (Tokyo, Japan), Thermo Fisher Scientific (Waltham, MA), and Sigma‐Aldrich (St. Louis, MO) produced all other reagents (ACS grade or higher) needed for synthesis. Steroid purification required silica columns (Yamazen Corp., Osaka, Japan) and thin‐layer chromatography plates (Bonna‐Agela Technologies Inc., Wilmington, DE). LC/MS grade methanol, acetonitrile, and formic acid was purchased from Thermo Fisher Scientific. XenoTech (Lenexa, KS) supplied pooled mixed gender human liver microsomes (Cat no. H0610, *n* = 50). Corning (Corning, NY) and Integrated DNA Technologies (Coralville, IA) manufactured the NADPH regeneration system and oligonucleotide primers, respectively. A QuikChange II Site‐Directed Mutagenesis Kit was purchased from Agilent (Santa Clara, CA) to produce aromatase variant overexpression vectors. The HEK293 cell line was procured from ATCC (Manassas, VA). G418, penicillin/streptomycin, fetal bovine serum, Opti‐MEM, and DMEM supplemented with 4.5 g/L glucose, 110 mg/L sodium pyruvate, and L‐glutamine was purchased from Invitrogen (Carlsbad, CA) along with an XCell electrophoresis system. Lipofectamine 2000, PVDF membranes, Pierce BCA protein assay kit, SuperSignal West Femto Maximum Sensitivity Substrate, sodium dodecyl sulfate (SDS), glycine, tris base, ammonium persulfate (APS), goat anti‐rabbit HRP‐conjugated antibody (cat. No. 31466), and tetramethylethylenediamine (TEMED) were also purchased from Thermo Fisher Scientific. Nonfat dry milk was prepared by BioRad (Hercules, CA). Sigma‐Aldrich (St. Louis, MO) supplied Ponceau staining solution, Tween 20, acrylamide/bis‐acrylamide solution, 2‐mercaptoethanol, estrone, androstenedione substrate, and estrone‐2,3,4‐^13^C_3_. Rabbit monoclonal anti‐aromatase antibody (cat. no. ab124776) was purchased from Abcam (Cambridge, MA).

### Reference library synthesis

EXE and ten C6‐oxidized or C17‐reduced EXE analogs were resuspended in ethanol and stored at −80°C following synthesis at Washington State University (Spokane, WA). Previous studies provide detailed descriptions of the synthesis, purification, and NMR‐based identity verification of each compound (Buzzetti et al. 1993; Marcos‐Escribano et al. 2009; Platt et al. 2016; Vatèle 2007).

### Creation of aromatase‐overexpressing HEK293

Stable overexpression of wild type aromatase in HEK293 was driven by a pcDNA3.1/V5‐His‐TOPO mammalian expression vector as previously described (Sun et al. 2010). Constitutive overexpression vectors encoding common aromatase variants Thr201Met and Arg264Cys were produced via site‐directed mutagenesis using the wild‐type plasmid as template. Variant expression vectors were amplified in BL21 grown under ampicillin selection for 16 h at 37°C. Sanger sequencing was used to confirm successful mutagenesis. Lipofectamine 2000 was used to transfect HEK293 with variant overexpression plasmids. Transfected HEK293 were grown in high‐glucose DMEM containing 700 *μ*g/mL G418, 10% FBS, and penicillin/streptomycin for at least 3 weeks. The cells were then harvested by resuspension in PBS, lysed via 4 freeze‐thaw cycles, and centrifuged for 15 min at 13,200*g* at 4°C. Microsomes for each cell line were prepared from the supernatant through differential centrifugation (1 h, 34000*g*) in a chilled Beckman L7‐65 ultracentrifuge (Brea, CA), resuspended in PBS, and stored at −80°C. The relative expression of aromatase was quantitated in triplicate by subjecting 20 *μ*g of protein from each overexpressing cell line to SDS‐PAGE in a 10% tris‐glycine polyacrylamide gel. Following transfer to PVDF for 90 min at 30 V, the membrane was blocked overnight at 4°C in 5% nonfat dry milk, washed for 30 min in 0.1% Tween, and probed overnight with anti‐aromatase primary antibody (1:2500). The next day, the membrane was again washed for 30 min, and probed with HRP‐conjugated goat anti‐rabbit antibody (1:7500) for 1 h at ambient temperature. Following another 30 min wash, the blot was incubated with SuperSignal West Femto Maximum Sensitivity Substrate per the manufacturer instructions and imaged on a ChemiDoc Imager (BioRad, Hercules, CA). Image J software (NIH, Bethesda, MD) was used to measure band density while Ponceau staining was used to validate even loading between lanes.

### Anti‐aromatase Activity Assays

Per 50‐*μ*L reaction in PBS (pH 7.4), 5 *μ*mol/L androstenedione, a NADPH regeneration system (1.55 mmol/L NADP+, 3.3 mmol/L glucose‐6‐phosphate, 3.3 mmol/L MgCl_2_, 0.5 *μ*L of 40 U/mL glucose‐6‐phosphate dehydrogenase), and 15 *μ*g of microsomes from HEK293 overexpressing wild type or variant aromatase were individually incubated with varying concentrations of each steroid. The steroid concentrations used for anti‐aromatase activity assays are as follows: EXE (0.05‐15 *μ*mol/L), 17*β*‐DHE (0.05–30 *μ*mol/L), 17*α*‐DHE (100 *μ*mol/L), 6*α*‐spirooxiranandrosta‐1,4‐diene‐3,17‐dione (0.05–30 *μ*mol/L), 6*β*‐spirooxiranandrosta‐1,4‐diene‐3,17‐dione (0.05–40 *μ*mol/L), 6*α*‐methylandrosta‐1,4,6‐triene‐3,17‐dione (0.5–30 *μ*mol/L), 6*α*‐methylandrosta‐1,4‐diene‐3,17‐dione (0.2–30 *μ*mol/L), 6‐hydroxymethylandrosta‐1,4,6‐triene‐3,17‐dione (2.5–300 *μ*mol/L), 17*β*‐hydroxy‐6‐hydroxymethylandrosta‐1,4,6‐triene‐3‐one (100 *μ*mol/L), 6*α*/*β*‐hydroxy‐6*α*/*β*‐hydroxymethylandrosta‐1,4‐diene‐3,17‐dione (100 *μ*mol/L), and 6*α*/*β*,17*β*‐Dihydroxy‐6*α*/*β*‐hydroxymethylandrosta‐1,4‐diene‐3‐one (100 *μ*mol/L). Organic solvent comprised <1% of the total volume of each enzymatic incubation, which proceeded at 37°C for 2 h. Reactions were terminated with 50 *μ*l of ice cold acetonitrile and centrifuged at 4°C for 15 min at 13,200*g*. Supernatants were collected and spiked with 50 ng of estrone‐2,3,4‐^13^C_3_ as an internal standard. An incubation with microsomes derived from non‐transfected HEK293 was also performed to serve as a negative control. Aromatization catalyzed by wild‐type or variant aromatase was likewise monitored in the presence of vehicle rather than EXE or compounds from the reference library to reflect maximal uninhibited estrone formation. Estrone was measured using a novel 6‐min direct detection UPLC/MS/MS method on the Waters Acquity platform using m/z transitions 271.17→133.09 as a marker for estrone and 274.15→162 for estrone‐2,3,4‐^13^C_3_. Mobile phase (57% methanol in 0.1% formic acid) was infused isocratically from 0 to 4 min at a flow rate of 0.4 mL/min. The column was then washed with methanol for 1 min followed by 1 min of re‐equilibration with mobile phase. Cone and collision voltages were set at 35 V and 20 V respectively. Dwell time for both compounds was 0.1 sec. IC_50_ values from incubations with wild‐type aromatase were calculated for each compound in GraphPad Prism 6 (La Jolla, CA). One‐way ANOVA was used to compare the IC_50_ value for EXE incubated with wild type aromatase with IC_50_ values for EXE incubated with overexpressed aromatase allozymes.

### EXE Metabolite Identification

A 50‐*μ*l incubation containing 50 *μ*g of HLM in PBS (pH 7.4), 400 *μ*mol/L EXE, and an NADPH regeneration system was placed in a 37°C water bath for 4 h before termination with 50 *μ*L of cold acetonitrile. After a 15‐min refrigerated centrifugation at 13,200*g*, the supernatant was examined for phase I EXE metabolites. A 10‐min UPLC method was used to separate and detect EXE and the ten other reference compounds through multiple reaction monitoring with positive mode electrospray ionization on a Waters ACQUITY UPLC/MS/MS system (Milford, MA). The 1.7 *μ*m ACQUITY UPLC BEH C18 column (2.1 mm × 50 mm, Ireland) used for these analyses was protected by a 0.2 *μ*m in‐line filter. The UPLC gradient conditions used have previously been described (Platt et al. 2016). The fragmentation characteristics and retention time of suspected metabolite peaks were compared to compounds from the reference library.

## Results and Discussion

### Wild‐type aromatase inhibition by EXE and its metabolites

**Figure 1:**
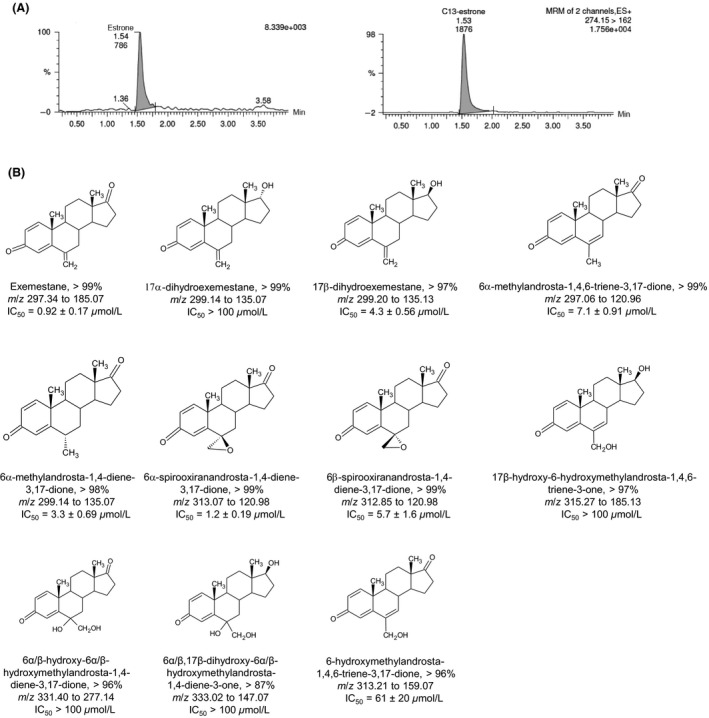
Results of anti‐aromatase activity assays using synthetic androgen inhibitors. (A) Chromatograms showing estrogen detection. Left, estrone; right, estrone‐2,3,4‐^13^C3 internal standard. (B) Chemical structures of species included in the synthesized reference library of EXE analogs. Percent purity is provided for each compound. Mass transitions used for UPLC/MS/MS‐based detection are listed, as well as IC_50_ values for wild‐type aromatase as determined by anti‐aromatase activity assay.

A reference library of purified androgens was assayed for in vitro inhibition of wild type aromatase by monitoring estrone formation (Fig. 1). In the present study, EXE (IC_50_ = 0.92 ± 0.17 *μ*mol/L) and its major metabolite 17*β*‐DHE (IC_50_ = 4.3 ± 0.56 *μ*mol/L) were potent and moderate inhibitors of aromatase respectively. These results agree with an earlier study which found that 17*β*‐DHE was approximately 2.6‐fold less potent than EXE (Buzzetti et al. 1993). Manufacturer data also references the diminished potency of C6‐oxidized or C17‐reduced EXE derivatives (Pfizer, 2016). Interestingly, the epoxide 6*α*‐spirooxiranandrosta‐1,4‐diene‐3,17‐dione was the most potent EXE analog assayed (IC_50_ = 1.2 ± 0.19 *μ*mol/L), exhibiting nearly 5‐fold more potency than its 6*β* stereoisomer (IC_50_ = 5.7 ± 1.6 *μ*mol/L). 17*α*‐DHE and three additional compounds exhibited negligible aromatase inhibition with IC_50_ values exceeding 100 *μ*mol/L (Fig. 1B). 6‐HME was determined to be 67‐fold less potent (IC_50_ = 61 ± 20 *μ*mol/L) than EXE in the present study, a large difference in potency also observed by Buzzetti et al. (1993). The remaining androgens assayed were 4–8‐fold less potent than EXE (IC_50_ = 3.3–7.1 *μ*mol/L). In keeping with the observations of Buzzetti et al. (1993), non‐epoxide C6‐oxidized metabolites exhibited minimal AAA.

### Impact of nonsynonymous polymorphisms on EXE potency

**Figure 2:**
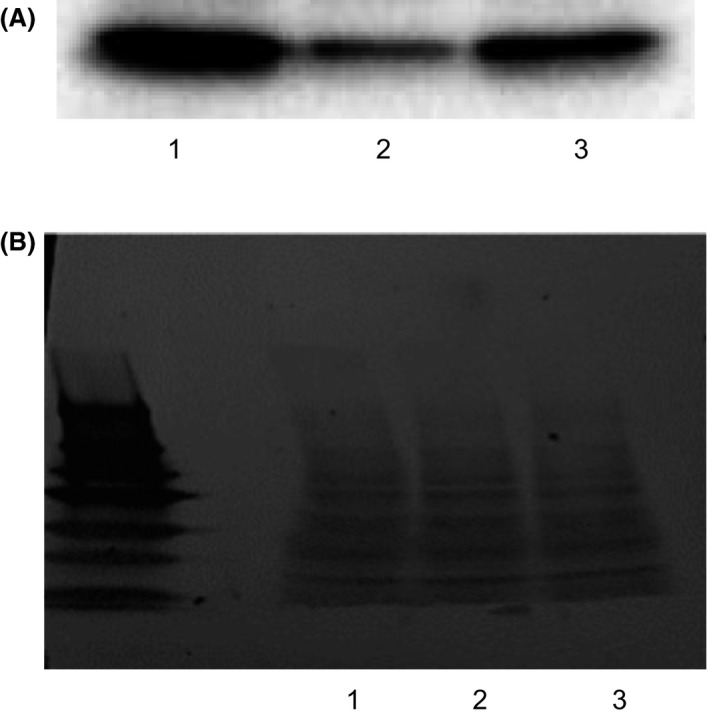
Relative quantification of overexpressed wild type and variant aromatase in HEK293 microsomes by Western blotting. (A) Lane 1, wild type aromatase; lane 2, aromatase^Thr201Met^; lane 3, aromatase^Arg264Cys^. Aromatase was detected using a monoclonal anti‐aromatase antibody (Abcam). (B) Ponceau total protein staining for aromatase normalization. Lanes correspond to the same three lanes described in panel A.

IC_50_ values describing EXE‐mediated aromatase inhibition did not significantly differ (*P* = 0.71) between wild type enzyme (0.92 ± 0.17 *μ*mol/L), aromatase^Thr201Met^ (0.86 ± 0.12 *μ*mol/L), and aromatase^Arg264Cys^ (0.97 ± 0.09 *μ*mol/L) in AAA assays normalized for relative aromatase expression (Fig. 2). Many aromatase polymorphisms exist, but data regarding the functional significance of variant alleles on human health is inconsistent (Baxter et al. 2001; Ma et al. 2005; Miyoshi et al. 2003). The prevalence of the Thr201Met allele is estimated as 5% in Caucasians and African Americans while the frequency of the Arg264Cys allele is 2.5% and 22.5% in Caucasian and African Americans respectively (Ma et al. 2005). One study of variant aromatase found that enzyme activity strongly correlated with expression levels in transiently transfected COS‐1 and further concluded that any differences from wild‐type in the overall activity of the Thr201Met and Arg264Cys allozymes are likely mediated by differential expression (Ma et al. 2005).

### EXE metabolite identification

**Figure 3:**
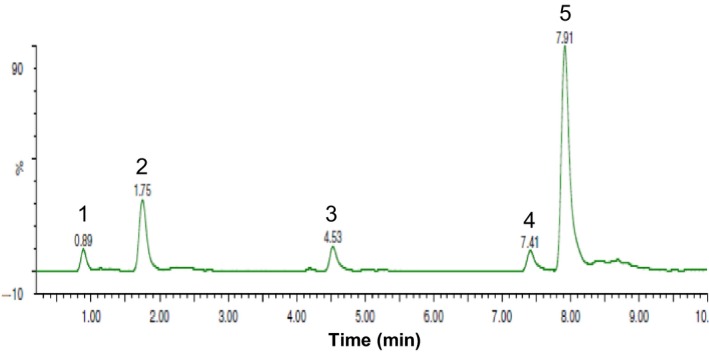
Identification of EXE metabolites in human liver microsomes. EXE metabolite profile was examined by UPLC/MS/MS after a 4 h incubation of pooled (*n* = 50) mixed gender human liver microsomes with EXE. Peak 1, 6*α*/*β*,17*β*‐dihydroxy‐6*α*/*β*‐hydroxymethylandrosta‐1,4‐diene‐3‐one; peak 2, 6*α*/*β*‐hydroxy‐6*α*/*β*‐hydroxymethylandrosta‐1,4‐diene‐3,17‐dione; peak 3, 6‐HME; peak 4, EXE; peak 5, 17*β*‐DHE.

17*β*‐DHE, 6‐HME, 6*α*/*β*‐hydroxy‐6*α*/*β*‐hydroxy‐methylandrosta‐1,4‐diene‐3,17‐dione, and 6*α*/*β*,17*β*‐dihydroxy‐6*α*/*β*‐hydroxymethyl‐androsta‐1,4‐diene‐3‐one were identified in incubations of EXE with pooled human liver microsomes through comparison to reference compounds (Fig. 3). Although we found four EXE metabolites, an in vitro study of EXE metabolism by Kamdem et al. (2011) detected six peaks corresponding to putative metabolites. Our assay was not designed to identify phase II metabolites suggesting that the two additional peaks observed in the previous study may correspond to conjugated metabolites, such as the 17*β*‐DHE‐glucuronide produced by UGT2B17 (Sun et al. 2010). Considering their low abundance and limited capacity to inhibit aromatase in our novel AAA assay, the three C6‐oxidized metabolites detected are unlikely to contribute to the overall pharmacology of EXE in vivo. However, these results show that 17*β*‐DHE is not only the predominant EXE metabolite formed in human liver microsomes, but also capable of inhibiting aromatase with moderate potency suggesting that it may make clinically relevant contributions to the overall response to EXE in women with ER+ breast cancer (Platt et al. 2016).

## Author Contribution

Participated in research design: Peterson and Lazarus. Conducted experiments: Peterson. Contributed new reagents or analytic tools: Xia, Chen, and Peterson. Performed data analysis: Peterson. Wrote or contributed to the writing of the manuscript: Peterson, Chen, Xia, and Lazarus.

## Disclosure

None declared.

## References

[prp2313-bib-0001] Baxter SW , Choong DY , Eccles DM , Campbell IG (2001). Polymorphic variation in CYP19 and the risk of breast cancer. Carcinogenesis 22: 347–349. 11181459 10.1093/carcin/22.2.347

[prp2313-bib-0002] Buzzetti F , Di Salle E , Longo A , Briatico G (1993). Synthesis and aromatase inhibition by potential metabolites of exemestane (6‐methylenandrosta‐1,4‐diene‐3,17‐dione). Steroids 58: 527–532. 8273115 10.1016/0039-128x(93)90029-m

[prp2313-bib-0003] Cavalcanti Gde A , Garrido BC , Leal FD , Padilha MC , de la Torre X , de Aquino Neto FR (2011). Detection of new urinary exemestane metabolites by gas chromatography coupled to mass spectrometry. Steroids 76: 1010–1015. 21530565 10.1016/j.steroids.2011.04.001

[prp2313-bib-0004] Cocchiari G , Allievi C , Berardi A , Zugnoni P , Strolin Benedetti M , Dostert P (1994). Urinary metabolism of exemestane, a new aromatase inhibitor, in rat, dog, monkey, and human volunteers. J Endocrinol Invest 17 (Suppl. 1 to no. 3): 78.

[prp2313-bib-0005] Coombes RC , Kilburn LS , Snowdon CF , Paridaens R , Coleman RE , Jones SE , et al. (2007). Survival and safety of exemestane versus tamoxifen after 2‐3 years' tamoxifen treatment (Intergroup Exemestane Study): a randomised controlled trial. Lancet 369: 559–570. 17307102 10.1016/S0140-6736(07)60200-1

[prp2313-bib-0006] Evans TR , Di Salle E , Ornati G , Lassus M , Benedetti MS , Pianezzola E , et al. (1992). Phase I and endocrine study of exemestane (FCE 24304), a new aromatase inhibitor, in postmenopausal women. Cancer Res 52: 5933–5939. 1394219

[prp2313-bib-0007] Hong Y , Yu B , Sherman M , Yuan YC , Zhou D , Chen S (2007). Molecular basis for the aromatization reaction and exemestane‐mediated irreversible inhibition of human aromatase. Mol Endocrinol 21: 401–414. 17095574 10.1210/me.2006-0281

[prp2313-bib-0008] Kamdem LK , Flockhart DA , Desta Z (2011). In vitro cytochrome P450‐mediated metabolism of exemestane. Drug Metab Dispos 39: 98–105. 20876785 10.1124/dmd.110.032276 PMC3014267

[prp2313-bib-0009] Ma CX , Adjei AA , Salavaggione OE , Coronel J , Pelleymounter L , Wang L , et al. (2005). Human aromatase: gene resequencing and functional genomics. Cancer Res 65: 11071–11082. 16322257 10.1158/0008-5472.CAN-05-1218

[prp2313-bib-0010] Marcos‐Escribano A , Bermejo FA , Bonde‐Larsen AL , Retuerto JI , Sierra IH (2009). 1,2‐Dehydrogenation of steroidal 6‐methylen derivatives. Tetrahedron 65: 7587–7590.

[prp2313-bib-0011] Miyoshi Y , Ando A , Hasegawa S , Ishitobi M , Yamamura J , Irahara N , et al. (2003). Association of genetic polymorphisms in CYP19 and CYP1A1 with the oestrogen receptor‐positive breast cancer risk. Eur J Cancer 39: 2531–2537. 14602139 10.1016/j.ejca.2003.08.017

[prp2313-bib-0012] Paridaens RJ , Dirix LY , Beex LV , Nooij M , Cameron DA , Cufer T , et al. (2008). Phase III study comparing exemestane with tamoxifen as first‐line hormonal treatment of metastatic breast cancer in postmenopausal women: the European Organisation for Research and Treatment of Cancer Breast Cancer Cooperative Group. J Clin Oncol 26: 4883–4890. 18794551 10.1200/JCO.2007.14.4659 PMC2652082

[prp2313-bib-0013] Pfizer (2016) Aromasin Exemestane Tablets.

[prp2313-bib-0014] Platt A , Xia Z , Liu Y , Chen G , Lazarus P (2016). Impact of nonsynonymous single nucleotide polymorphisms on in‐vitro metabolism of exemestane by hepatic cytosolic reductases. Pharmacogenet Genomics 26: 370–380. 27111237 10.1097/FPC.0000000000000226 PMC5088049

[prp2313-bib-0015] Sun D , Chen G , Dellinger RW , Sharma AK , Lazarus P (2010). Characterization of 17‐dihydroexemestane glucuronidation: potential role of the UGT2B17 deletion in exemestane pharmacogenetics. Pharmacogenet Genomics 20: 575–585. 20697310 10.1097/FPC.0b013e32833b04af PMC3076703

[prp2313-bib-0016] Thompson EA Jr , Siiteri PK (1974). Utilization of oxygen and reduced nicotinamide adenine dinucleotide phosphate by human placental microsomes during aromatization of androstenedione. J Biol Chem 249: 5364–5372. 4153532

[prp2313-bib-0017] Traina TA , Poggesi I , Robson M , Asnis A , Duncan BA , Heerdt A , et al. (2008). Pharmacokinetics and tolerability of exemestane in combination with raloxifene in postmenopausal women with a history of breast cancer. Breast Cancer Res Treat 111: 377–388. 17952589 10.1007/s10549-007-9787-1

[prp2313-bib-0018] Uhlén M , Fagerberg L , Hallström B , Lindskog C , Oksvold P , Mardinoglu A , et al. (2015). The human protein atlas. http://www.proteinatlas.org (last accessed April 2017).

[prp2313-bib-0019] Vatèle J (2007). 2‐(Prenyloxymethyl)benzoyl (POMB) group: a new temporary protecting group removable by intramolecular cyclization. Tetrahedron 63: 10921–10929.

